# Factors affecting utilization of sexual and reproductive health services among women with disabilities- a mixed-method cross-sectional study from Ilam district, Nepal

**DOI:** 10.1186/s12913-021-07382-4

**Published:** 2021-12-23

**Authors:** Rupa Shiwakoti, Yogendra Bahadur Gurung, Ram Chandra Poudel, Sandesh Neupane, Ram Krishna Thapa, Sailendra Deuja, Ram Sharan Pathak

**Affiliations:** 1 Central Department of Population Studies, Tribhuvan University, Kirtipur, Kathmandu, Nepal; 2 Country Coordinating Mechanism Nepal, Teku, Kathmandu, Nepal; 3 Health Inspector, Health Section, Nagarjuna Municipality, Kathmandu, Nepal; 4 International Humanitarian Action, University of Warsaw, Warsaw, Poland; 5 Karuna Foundation Nepal, Central Office, Kathmandu, Nepal

**Keywords:** Women with disabilities, Sexual and reproductive health, Utilization, Health belief model, Ilam, Nepal

## Abstract

**Background:**

Persons with disabilities can have physical, mental, intellectual, or sensory impairments which can hinder their social participation. Despite Sustainable Development Goals call for “universal access to sexual and reproductive health (SRH)”, women with disabilities (WwDs) continue to experience barriers to access SRH services in Nepal. This study evaluated factors affecting the utilization of SRH services among WwDs in Ilam district, Nepal.

**Methods:**

A mixed-method study with 384 WwDs of reproductive age was conducted in Ilam district, eastern Nepal. Quantitative data were collected using a structured questionnaire. Relationships between utilization of SRH services and associated factors were explored using multivariate logistic regression analysis. Qualitative data were collected from focus groups with female community health volunteers and interviews with WwDs, health workers and local political leaders. They were audio-recorded, translated and transcribed into English and were thematically analyzed.

**Results:**

Among 384 respondents (31% physical; 7% vision,16% hearing, 7% voice&speech,12% mental/psychosocial, 9% intellectual, 18% multiple disabilities), only 15% of them had ever utilized any SRH services. No requirement (57%) and unaware of SRH services (24%) were the major reasons for not utilizing SRH services. A majority (81%) of them reported that the nearest health facility was not disability-inclusive (73%), specifically referring to the inaccessible road (48%). Multivariate analysis showed that being married (AOR = 121.7, 95% CI: 12.206–1214.338), having perceived need for SRH services (AOR = 5.5; 95% CI: 1.419–21.357) and perceived susceptibility to SRH related disease/condition (AOR = 6.0; 95% CI:1.978–18.370) were positively associated with the utilization of SRH services. Qualitative findings revealed that illiteracy, poor socioeconomic status, and lack of information hindered the utilization of SRH services. WwDs faced socioeconomic (lack of empowerment, lack of family support), structural (distant health facility, inaccessible-infrastructure), and attitudinal (stigmatization, bad behaviour of health care providers, perception that SRH is needed only for married) barriers to access SRH services.

**Conclusions:**

Utilization of SRH services among WwDs was very low in Ilam district, Nepal. The findings of this study warrant a need to promote awareness-raising programs to WwDs and their family members, sensitization programs to health service providers, and ensure the provision of disability-inclusive SRH services in all health facilities.

**Supplementary Information:**

The online version contains supplementary material available at 10.1186/s12913-021-07382-4.

## Background

Persons with disabilities are “those who have long-term physical, mental, intellectual, or sensory impairments which, in interaction with various barriers, may hinder their full and effective participation in society on an equal basis with others” [1]. According to World Health Organization (WHO), about 15% of the world’s population live with some form of disability, of whom two to four% experience significant difficulties in functioning [2]. The great majority of persons with disabilities are part of 80% of the world’s population that live in developing countries [3]. In Nepal, the prevalence of persons with disabilities is 1.94% and almost half of them are women [4].

Sexual and Reproductive Health (SRH) is an essential component of health and one of the pillars for sustainable development. SRH services are necessary for all people including persons with disabilities, both married and unmarried. All too often, the SRH of persons with disabilities has been overlooked by the disability community as well as those working on SRH. Persons with disabilities have the same needs for SRH services as the rest of the general population. Persons with disabilities may have greater needs for SRH education and care than their counterparts due to existing barriers and their increased vulnerability to abuse. Research shows that persons with disabilities are as sexually active as persons without disabilities. Despite this, too often their sexuality has been ignored and their reproductive rights denied [5].

Access to SRH services is essential for people with disabilities. Growing evidence in North America, Europe and Australia indicate that women with disabilities have poor pre-conception health status (diabetes, frequent mental distress, obesity, asthma, lack of emotional support) [6]; and have a greater risk for unplanned or unintended pregnancies [7]. They also can have perinatal and postnatal complications (gestational diabetes, hypertension, urinary tract infection, preterm rupture of membranes, cesarean delivery, and postpartum depression) [7–9] and adverse pregnancy outcomes (small for gestation, preterm birth, with smaller numbers of studies revealing elevated risk for other adverse neonatal and infant outcomes) [10].

Despite Sustainable Development Goals (SDGs) explicit call for ensuring “universal access to sexual and reproductive health and reproductive rights” and specific articles in the United Nations Convention on the Rights of Persons with Disabilities (CRPD) [1], the SRH of many women with disabilities continue to be unattainable and little research has examined the specific barriers these women face in developing countries.

Nepal has a National Guideline for Disability Inclusive Health Services 2019 [11] which guides how to mainstream disability inclusion in health service delivery, and how health providers can operationalize their responsibilities under disability-related laws and policies. It focuses on providing disability-inclusive health services i.e. ensuring people with disabilities have the same rights, participation and inclusion in health services as the general population by adjusting the health system for reasonable accommodations and educating health workers for positive attitudes and behaviours [11]. However, its weak enforcement persists which echoes with broader studies [12, 13] related to implementation problems in Nepal’s health system.

There is very little data available on the SRH of women with disabilities in developing countries like Nepal. Though there are many surveys and studies on SRH of women in Nepal, the majority of them have not disaggregated their data by disability. A cross-sectional study conducted in six (out of 76) districts of Nepal showed that 76% of young persons with disabilities had knowledge of major components of SRH such as family planning, safe abortion and sexually transmitted infections (STIs). About 22% of the young persons with disabilities reported difficulty in communicating with the SRH service provider [14].

The utilization of SRH services among women with disabilities and its associated factors were inadequately explored in Nepal. There is an urgent need to identify these barriers in Nepal’s health service context. Thus, this study was carried out with the main objective to assess the utilization of SRH services among women with disabilities and explore factors affecting it in Ilam district, Nepal.

## Methods

### Study site

The study was conducted in Ilam, a hilly district situated in province number one of eastern Nepal with an area of 1703 km^2^ and 290,254 population. It consists of six rural municipalities and four urban municipalities. Ilam district was selected purposively for the study as the prevalence of disability is 2.63% in Ilam, which is higher than the national disability prevalence rate of 1.94 [4]. Furthermore, the researcher of this study was familiar with the study area being a former employee of Karuna Foundation Nepal [15], a non-government organization working in Ilam.

### Study design

The study employed a mixed-method study design, with a cross-sectional survey in the quantitative arm and semi-structured interviews and focus group discussions in the qualitative arm. The study was conducted between September 2018 and February 2020.

### Sampling

The sample size was calculated using Epi Info version seven. Since the prevalence of SRH services utilization among women with disabilities was not available, it was considered to be 0.5. Furthermore, with a margin of error of 5 % at a 95% confidence level, the estimated sample for the quantitative study was 384.

A complete list of women with disabilities [16] aged 15–49 years residing in Ilam district was prepared with the help of Women and Child Officers, the Government of Nepal, who issue disability ID cards. Along with the administrative data, female community health volunteers [17] and community-based rehabilitation facilitators, who work at the community level, were approached to identify missing women with disabilities. From the sampling framework of 829 women with disabilities aged 15–49 years, 384 respondents were selected using systematic random sampling. The inclusion criteria were as follows:

1. Women of reproductive age (15–49 years),
2. Had disability ID card or in the process of issuing ID card, and
3. Fell under the disability category of the “Washington Group Short Set of the questionnaire” [18]
  The “Washington Group Short Set of the questionnaire” [18] was used to confirm disability which contains six questions focusing on six core functional domains: seeing, hearing, walking, cognition, self-care and communication. Following are the questions:
  1. Do you have difficulty seeing, even if wearing glasses?
  2. Do you have difficulty hearing, even if using a hearing aid?
  3. Do you have difficulty walking or climbing steps?
  4. Do you have difficulty remembering or concentrating?
  5. Do you have difficulty (with self-care such as) washing all over or dressing?
  6. Using your usual (customary) language, do you have difficulty communicating, for example, understanding or being understood?

Each respondent was requested to respond on the following response scales:

- No - no difficulty
- Yes – some difficulty
- Yes – a lot of difficulties
- Cannot do at all

Only those respondents who replied “Yes, a lot of difficulties” or “Cannot do it at all” in at least one domain of the Washington Group Short Set questionnaire [18], were included in the study. If the selected respondent did not fall under the disability category, the subsequent sample from the sampling framework was selected.

Disability was grouped based on the disability ID card. For those without a disability ID cards, disability was grouped with the support of community-based rehabilitation facilitators. As per the Nepal disability rights act 2017 [16], disabilities are categorized into ten types and four categories based on their severity. An additional file shows this in more detail (see Additional file 1).

Among 384 respondents, one-third (31%) of them had a physical disability, 7 % had disability-related to vision, 16% had disability-related to hearing, 7 % had disability-related to voice and speech, 12% had mental/psychosocial disability, 9 % had intellectual disability, and 18% had multiple disabilities. Among 318 disability ID cardholders, 14, 40, 25 and 21% of them had profound, severe, moderate and mild disabilities respectively (Table 1).

**Table 1:** Disability-related characteristics of respondents (*n* = 384)

Variables	Frequency	Per cent
**Types of disability**^a^		
Physical disability	119	31.0
Disability-related to vision-blindness	7	1.8
Disability-related to vision-low vision	11	2.9
Disability-related to vision -complete blind	8	2.1
Disability-related to hearing -deaf	33	8.6
Disability-related to hearing -hard of hearing	29	7.6
Disability-related to voice and speech	26	6.8
Mental or psychosocial disability	44	11.5
Intellectual disability	35	9.1
Disability-related to Autism	2	0.5
Multiple disabilities	70	18.2
**The severity of disability (*n*** **= 318)**^a^		
Profound	45	14.2
Severe	127	39.9
Moderate	80	25.2
Mild	66	20.8

^a^Based on the type and severity of disability defined by the Government of Nepal [16]

For the qualitative study, focus groups with female community health volunteers, in-depth interviews with women with disabilities, semi-structured interviews with health workers (providing basic health services including SRH services in local government health facilities) and local political leaders (elected chairperson of local government) were conducted. The samples for the qualitative study were selected purposively.

### Data collection tools

For the quantitative study, a structured questionnaire was developed based on the study objectives and variables in the guidance of subject experts. The questionnaire included information related to sociodemographic and socioeconomic characteristics, type and severity of disability [16], women empowerment [19], knowledge and attitude on SRH [20], access to SRH services, provision of disability-inclusive SRH services [11]), and utilization of SRH services. The questionnaire was digitalized in tablets using Commcare [21] software. Pre-testing of the tools was done in Sunsari, another district of province number one of eastern Nepal. Some reordering of questions was done after the pre-test.

For the qualitative study, a codebook guide was prepared by adopting the Health Belief Model [22]. Interview guidelines were developed to generate information related to perception on disability, the need for SHR services by women with disabilities, availability of SRH services, utilization of SRH services by women with disabilities and challenges faced to improve its utilization. Themes within the guides were discussed among the authors and piloted with participants. The thematic guide was amended based on their feedback and was adapted for the interview and focus group by interviewers. Following an approach previously used [23], the thematic guide for this study outlined major themes and sample questions that allowed an interviewer to adapt and use them for interviews and focus groups.

### Data collection technique

Face-to-face interviews of women with disabilities were conducted to collect quantitative data using a pretested structured questionnaire. Support from the caretaker was sought for those who were unable to respond (27%). A total of 10 public health undergraduates were trained as enumerators. Data collection was completed over 2 months starting from 23rd May to 21st July 2019.

Qualitative data were collected from semi-structured interviews and focus groups to supplement the findings, facilitate triangulation as well as explore in detail the underlying elements that might have affected the service utilization from both the women with disabilities and the service providers’ perspectives. The interviews were taken by two teams each comprising of a lead female interviewer and a female note taker. They were public health undergraduates, trained in qualitative study and familiar with the local language and cultural context. All interviews and focus groups were conducted with the help of a codebook guide prepared by adopting the Health Belief Model [22].

The list of the participants for the semi-structured interview and focus groups is mentioned below:

1. Two women with disabilities,
2. Two health workers (one male and one female) providing basic health services including SRH services in the local government health facility
3. Two elected chairpersons (male) of local government
4. Two focus groups with female community health volunteers (11 respondents in group one and eight in group two)

The respondents for the qualitative study were selected purposively. None of the respondents refused to participate and there were no dropouts. Focus groups with female community health volunteers were conducted in a separate hall of the health post. While the in-depth interviews of women with disabilities were conducted in a separate room of their house, interviews of health workers were conducted in a quiet room of the health post where they work. Likewise, the interviews of the local political leaders were conducted at their offices in a preferred/quiet room. Permissions were taken from the participants for the recordings. An audio recording was done with an audio recorder and field notes were taken during the interviews and focus groups. It took 30–45 min for the focus groups and 15–20 min for other interviews. Sampling for qualitative study was stopped once saturation level [23] was met and no additional information was derived from the interviews. All recorded interviews and focus groups were later transcribed and translated verbatim into English by researchers. Transcripts were returned to the selected respondents for comment and/or correction but no change was suggested by them.

### Ethical approval

Ethical approval was obtained from Nepal Health Research Council [24]. Approval was also obtained from the health office, urban and rural municipalities of Ilam district. Information was provided on potential risk, discomfort and benefits to the respondents, and confidentiality, the rights to refuse or withdraw, and the rights to information. The interviewer gave a self-introduction and briefed about the study before starting the interview/focus group. Written informed consent was obtained from the respondents. For the respondents below 18 years of age, written consent from their guardian and accent from the respondents were taken. To protect the confidentiality of respondents, interviews were taken in a quiet room (one-on-one), permission was taken for recording the interview, records were secured through the use of password protected files and codes were used to link respondents’ responses. Anonymization was done to avoid the personal identifiers for all the respondents. Each respondent was provided with a temporary ID number.

### Analysis

For the quantitative study, data was analyzed using Statistical Package for Social Science (SPSS) software [25] version 23.

Utilization of SRH was the dependent variable and was defined as the use of any of the following SRH services: maternal and newborn care; contraceptive information and services; prevention and appropriate treatment of infertility; safe abortion and post-abortion care; combatting HIV/AIDS and other sexually transmitted diseases; prevention of gender-based violence, care for victims and information, education and counselling on sexual violence; actions to eliminate harmful traditional practices such as early and forced marriage; and comprehensive sexuality education and youth-friendly services.

Independent variables of the study consisted of sociodemographic characteristics (age, religion, ethnicity, educational status, marital status, type of family, household size), socioeconomic characteristics (occupation, wealth index constructed by principal component analysis), disability (type and severity of disability [16]), women empowerment [19] (involvement in household decision making, membership in community group, cash earning, ownership of house/land and educational status), knowledge and attitude on SRH [20], and access to SRH services (time taken to reach the nearest health facility, distance between home and nearest health facility, enrollment to health insurance, media exposure and provision of disability-inclusive SRH services [11]).

Wealth index was measured as a composite indicator of economic status constructed by principal component analysis of more than fifty variables including household related variables such as main fuel for cooking, type of toilet facility, the main source of drinking water, possession of land, the main material of wall, roof and floor; some household assets such as radio, television, computer, mobile, landline phone, refrigerator, sofa, cupboard, fan, motorcycle, cow/bull/buffalo, horse/donkey/mule, goat, pig/wild boar and hen/duck. The wealth quintiles (from lowest to highest) are ranked into five equal categories, each comprising 20% of the study population.

Women’s empowerment was assessed using the Women’s Empowerment Index (WEI) [19], which was composed of five variables. Following is the definition and scoring of the five indicators been used in the development of the WEI:

- Involvement in household decision-making
  This indicator included three decisions: access to health care, household purchasing and freedom to visit relatives. The responses were coded into three categories. Those respondents who participated in all three decisions received a “two” score, those who participated in one or two decisions received a “one” score, and those who did not participate in any decisions received a “zero” score.
- Membership in community groups
  Those respondents who were a member of any community group, such as a mothers’ group, saving group, women’s group and others, were scored as “one” and those who were not involved in any group, were scored as “zero”.
- Cash earnings
  Those respondents who earned cash only or both cash and in-kind were given a score of “one” and those who did not earn cash at all were given a score of “zero”.
- Ownership of house/land
  Those respondents, who owned a house, land, or both alone or jointly with their husband, received a score of “one” and those who did not own any house, land or both, received a score of “zero”.
- Education
  It was measured on an ordinal scale under the following categories: No education (illiterate and informal education), primary level (up to grade five), some secondary level (from grade six to ten), and higher secondary and above.
  Those respondents who have attained secondary or higher education were scored “two”. Those respondents who have attained primary level education were scored “one” and those who did not attend school at all were scored “zero”.

The total scores were ranged from zero to seven. Those respondents who received one to two scores in aggregate were grouped in the low empowerment level. Those respondents who scored three or four were categorized as moderately empowered and those who scored five to seven were categorized as highly empowered.

Knowledge of SRH was assessed using composite indicators adapted from Measure Evaluation for Sexual and Reproductive Health Knowledge [20] comprising 16 questions as follows (Table 2).

**Table 2:** Composite indicator of knowledge on sexual and reproductive health

S.N.	Questions	Score
1	Heard about SRH	1
2	Know about the components of SRH	8
3	Know about the most fertile period in a woman’s ovulatory cycle	1
4	Know that woman may conceive before resuming her period after delivery	1
5	Heard about STI/HIV	1
6	Know the modes of transmission of HIV	5
7	Know that STI/HIV can be transmitted through single unsafe sexual contact with a person having STI/HIV	1
8	Know the sign and symptoms of STI	5
9	Accepts that a healthy-looking person can have HIV	1
10	Know about the preventive measures of STI	4
11	Heard about HIV testing	1
12	Know about family planning	1
13	Know any modern contraceptive method	1
14	Know abortion is legal in Nepal	1
15	Know the circumstances allowing legal abortion	4
16	Availability of safe abortion services	1
**Total Score**		**37**

The total score attained by each respondent was converted into a percentage. The level of knowledge was determined based on the following scoring mentioned below (Table 3).

**Table 3:** Scoring of knowledge on sexual and reproductive health

Knowledge on SRH	Scoring
Very good	80% and above
Good	60–79%
Satisfactory	41–59%
Poor	21–40%
Very Poor	20% and below

For the quantitative study, a cross-tabulation using the Chi-square test (or Fisher’s exact test) was carried out to check for associations between dependent and independent variables. Multivariate analysis was carried out for those variables, which were significant (*p* < 0.05) at the 95% confidence interval (CI) in the bivariate analysis after checking collinearity.

For the qualitative study, the data were coded by two female researchers. Data collected from interviews and focus groups underwent thematic analysis using the codebook approach [26]. The Health Belief Model [22] was adopted and the themes were identified in advance from the model. Data were allocated to the predetermined themes using the codebook as the guide. First, using a deductive approach, data were categorized based on the themes for the interview/focus group guides; additional categories and themes were added, inductively, to incorporate the emerging themes from the transcripts [27]. Final themes were discussed among the authors and were later categorized into main themes and sub-themes. Board themes and sub-themes form the basis of the result section. Feedback from respondents on the research findings was sought and incorporated to ensure that their meanings and perspectives were represented. Adhering to a principle of triangulation [28], a mix of balance between delineating the differences and common perspectives between four various respondents are presented under each theme. Excerpts were chosen based on their relevance to the themes in addition to the recurrences and uniqueness. The qualitative study follows a standard consolidated criterion for reporting qualitative studies (COREQ) [16] guideline.

## Results

The median age of the respondent was 35 years. Among 384 respondents, one-third (35%) of them could not read/write. More than half of them were Hindu (56%), never married (64%), belonged to a joint family (59%) and were unemployed (59%). The average household size was 5.1. About one in seven (15%) had ever utilized any SRH services.

Age, type of family, family size, marital status, educational status of the study population and their caretaker were significantly associated with the utilization of SRH services. Women aged 35 and above (OR = 3.4, CI: 1.795–6.430), belonged to the joint family (OR = 2.0, CI:1.100–3.760), had less than five family sizes (OR = 2.3, CI:1.304–4.011), could read/write (OR = 2.3, CI:1.201–4.612), had caretakers who could read/write (OR = 2.9, CI:1.009–8.588), and ever married (OR = 174.7, CI:23.818–1281.599) were more likely to utilize SRH services compared to their counterparts (Table 4).

**Table 4:** Sociodemographic and socioeconomic characteristics of respondents and their association with utilization of SRH services (*n* = 384)

Characteristics	Utilization of SRH services		***p***-value	Crude OR (95%CI)
	Yes n (%)	No n (%)		
**Age**				
35 and above	45(22.2)	158(77.8)	0.000*	3.4(1.795–6.430)
Below 35	14(7.7)	167(92.3)		Ref
**Religion**				
Hindu	39(18.3)	174(81.7)	0.076	1.7(0.946–3.027)
Buddhist/Kirat/ Christian	20(11.7)	151 (88.3)		Ref
**Ethnicity**^**a**^				
Brahman/Chhetri	24(19.5)	99(80.5)	0.124	1.2(0.885–2.770)
Dalit/Janajati/Madhesi/Thakuri/Dasnami	35(13.4)	226(86.6)		Ref
**Type of family**				
Joint	43 (18.9)	185(81.1)	0.024*	2.0(1.100–3.760)
Nuclear	16(10.3)	140 (89.7)		Ref
**Household size**				
Less than 5	33(22.1)	116(77.9)	0.004*	2.3(1.304–4.011)
5 or more	26(11.1)	209(88.9)		Ref
**Educational status**				
Literate	47(18.8)	203(81.2)	0.013*	2.3(1.201–4.612)
Cannot read/write	12(9.0)	122(91.0)		Ref
**Educational status of caretaker (*n*** **= 314)**				
Literate	34(14.2)	205(85.8)	0.048*^#^	2.9(1.009–8.588)
Cannot read/write	4(5.3)	71(94.7)		Ref
**Marital Status**				
Ever married	58(41.7)	81(58.3)	0.000*^#^	174.7(23.818–1281.599)
Never married	1(0.4)	244(99.6)		Ref
**Occupation**				
Employed	35(29.4)	84(70.6)	0.000*	4.2(2.353–7.441)
Unemployed	24(9.1)	241(90.9)		Ref
**Wealth quintile**				
Lowest	11(14.5)	65(85.5)	0.810	1.1(0.537–2.217)
Other	48(15.6)	260 (84.4)		Ref

^a^ As per the classification system used by the Health Management Information System section of the Department of Health Services, Nepal [29]

The crude odds ratio is the odds ratio that identifies the association between variables with the use of SRH services. The variable for which *p*-value is less than 0.05(*) is considered significant

^#^Fisher’s exact test

*Ref* reference group

### Contributing factor for the utilization of SRH services: socioeconomic status

Though socioeconomic status was not found to be significantly associated with the utilization of SRH services, the qualitative finding showed that poor socioeconomic status was one of the barriers to the utilization of SRH services among women with disabilities. Socioeconomic status was linked with education and access to information. Those women with disabilities who belonged to families with poor socioeconomic status were deprived of education and knowledge on SRH, which ultimately must have led to less utilization of SRH services.

*“Women with disabilities who belong to rich families are more educated and aware of SRH services than those who belong to poor families. Poor economic conditions and illiteracy are barriers to utilizing SRH services. (participant number 4, local political leader during KII)”*

### Utilization of SRH services

The utilization of SRH services among women with disabilities was low (15%). Only 12% had ever received maternal and newborn care, 11% had ever utilized contraceptive information and services, 0.3% had ever received prevention and appropriate treatment of infertility services, and 0.5% had ever utilized safe abortion and post-abortion care. None of them had ever utilized other SRH services related to HIV/AIDS and other sexually transmitted diseases; gender-based violence, elimination of harmful traditional practices such as early and forced marriage; and comprehensive sexuality education and youth-friendly services. No requirement (57%) and unaware of SRH services (24%) were the major reasons for not utilizing SRH services.

Among 384 respondents, 121 (32%) had ever experienced pregnancy and childbirth. Among them, only half (51%) had received antenatal checkups, more than two-thirds (67%) had delivered their child at home mainly with support from family members (52%). Moreover, only 34% of them had received postnatal care (Table 5).

**Table 5:** Utilization of SRH services among women with disabilities (*n* = 384)

Variables	Frequency	Per cent
**Utilization of SRH services**		
Yes	59	15.4
No	325	84.6
**Utilization of SRH services by component**^**a**^		
Maternal and newborn care services	45	11.7
Contraceptive information and services	38	9.8
Prevention and appropriate treatment of infertility	1	0.3
Safe abortion and post-abortion care services	2	0.5
**Reason for not utilizing the service from the nearest health facility (*n*** **= 325)**		
No need	186	57.2
Facility too far away	21	6.5
Health facility is not disability inclusive	27	8.3
Don’t know about the service	78	24.0
Other	13	3.4
**Had experience of pregnancy and childbirth**		
Yes	121	31.6
No	263	68.4
**Received antenatal check-up in the last pregnancy(*n*** **= 121)**		
Yes	44	36.0
No	77	64.0
**Place of delivery (*n*** **= 121)**		
Health facility	40	33.1
At home	80	66.1
On the way	1	0.8
**Assisted the delivery (*n*** **= 121)**		
Formal health worker	37	30.6
Female community health volunteers	4	3.3
Family members	63	52.1
Relative/friends	5	4.1
Traditional Birth Attendant	7	31.5
**Received postnatal care after the last delivery (*n*** **= 121)**		
Yes	41	33.9
No	80	66.1

^a^ Multiple responses

Others: Providers are often unavailable, prefer to receive care at home, have no one available to accompany, no quality services, language barrier, scared of side effects, no tradition

### Disability and utilization of SRH services

Both type and severity of disability were significantly associated with the utilization of SRH services. Those women with physical disabilities (OR = 3.0; CI:1.692–5.254) and mild disability (OR = 2.8; CI: 1.500–5.262) were three times more likely to utilize SRH services compared to their counterparts (Table 6).

**Table 6:** Disability-related characteristics of respondents and their association with utilization of SRH services (*n* = 384)

Characteristics	Utilization of SRH services		***p***-value	Crude OR (95%CI)
	Yes n (%)	No n (%)		
**Type of disability**				
Physical disability	31(26.1)	88(73.9)	0.000*	3.0(1.692–5.254)
Other than physical disability^a^	28(10.6)	237(89.4)		Ref
**The severity of disability**^**b**^ **(*n*** **= 318)**				
Mild	19(28.8)	47(71.2)	0.001*	2.8(1.500–5.262)
Moderate/Severe/Profound	40(2.6)	278(87.4)		Ref

^a^ Disability-related to vision (26), hearing (62), voice and speech (26), mental/psychosocial disability (44), intellectual disability (35), Autism (2) and multiple disabilities (70)

^b^Severity of disability was determined based on disability ID card issued by the Government of Nepal as per Nepal Disability Rights Act 2017 [16]

The crude odds ratio is the odds ratio that identifies the association between variables with the use of SRH services. The variable for which *p*-value is less than 0.05(*) is considered significant

*Ref* reference group

### Socioeconomic barriers to the utilization of SRH service: lack of family support

Family support is essential for all including persons with disabilities. The qualitative findings pointed out the need for assistance to women with disabilities but their family members were found to be often occupied in their household chores and livelihood activities. Lack of support from family members was reported as a barrier to the utilization of SRH services among women with disabilities.

*“Those women who have a mild physical disability can utilize SRH services. But those who have mental disabilities and severe forms of other disabilities are unable to utilize such services. They need someone to escort but their family members are often busy in household chores and livelihood related activities (participant number 3, local political leader during interview)”*

### Empowerment and utilization of SRH services

Among 384 respondents, about two-thirds (61%) did not participate at all in three key household decisions (health care, major household purchases and visit to family or relatives). Only half of them (52%) were members of the community groups. Less than a quarter (22%) earned cash or in-kind and only 12% owned any house or land either alone or jointly with someone else.

About two-thirds (63%) of them were less empowered, a quarter (26%) were moderately empowered and only 11% were highly empowered.

Those women with disabilities who participated in the household decisions (OR = 6.1; CI: 3.272–11.545), were a member of the community group (OR = 2.4; CI: 1.320–4.224), earned cash/in-kind (OR = 8.6; CI: 4.707–15.745), and were moderately/highly empowered (OR = 4.5; CI: 2.471–8.101) were more likely to utilize SRH services compared to their counterparts (Table 7).

**Table 7:** Empowerment level of respondents and their association with utilization of SRH services (*n* = 384)

Characteristics	Utilization of SRH services		***p***-value	Crude OR (95%CI)
	Yes n (%)	No n (%)		
**Involvement in household decision making**				
Participate in all decisions	44(29.5)	105(70.5)	0.000*	6.1(3.272–11.545)
No participation	15(6.4)	220(93.6)		Ref
**Membership in the community group**				
Yes	39(21.0)	147(79.0)	0.004*	2.4(1.320–4.224)
No	20(10.1)	178(89.9)		Ref
**Earn cash or in-kind**				
Yes	36(41.9)	50(58.1)	0.000*	8.6(4.707–15.745)
No	23(7.7)	275(92.3)		Ref
**Ownership of house/land**				
Yes	10(21.7)	36(78.3)	0.205	1.6(0.764–3.515)
No	49(14.5)	289(85.5)		Ref
**Women empowerment**^a^				
Moderately and highly empowered	40(27.8)	104(72.2)	0.000*	4.5(2.471–8.101)
Low empowered	19(7.9)	221(92.1)		Ref

The crude odds ratio is the odds ratio that identifies the association between variables with the use of SRH services. The variable for which *p*-value is less than 0.05(*) is considered significant

*Ref* reference group

^a^Women’s empowerment was a composite index of women empowerment comprising involvement in household decision-making, membership in the community group, cash earning, ownership of house/land and educational status of women [30]

### Socioeconomic barriers to access of SRH service: lack of empowerment

The qualitative findings showed consistency with the quantitative findings revealing the lack of empowerment as a barrier to access SRH services by women with disabilities. The study showed that women with disabilities were not empowered enough to express their SRH needs or problems. Moreover, they were also prone to violence.

*“Women with disabilities themselves are not empowered enough to share their SRH needs or problems. If someone misbehaved, other women could discuss it openly and say ‘No’. But, in regards to women with disabilities, they are not empowered. They are unable to raise their voice and defend themselves. (participant number 4, local political leader during interview)”*

The qualitative findings also highlighted the issues of sexual violence and forced control on reproduction among women with disabilities.

*“Many women with disabilities are not getting any support from home and they are not aware of SRH. And (sigh) those women with disabilities, who are bedridden, are prone to sexual violence. There are some instances of rape and forced marriage among women with disabilities in our locality. (participant number 2, woman with disability during in-depth interview)”*

*“We found that some family members are providing Depo-Provera injection to their daughters with disabilities. The family members have to go outside for work. They feel that girls with disabilities are prone to sexual violence in their absence. So, they provide Depo-Provera injection to prevent them from being pregnant. (participant number 7, female community health volunteer during focus group)”*

### Media exposure and utilization of SRH services

The media exposure of women with disabilities seemed quite low. Those respondents who often listened to radio/FM were two times more likely to utilize SRH services compared to their counterparts (OR = 2.3; CI: 1.303–4.088) (Table 8).

**Table 8:** Media Exposure and their association with utilization of SRH services (*n* = 384)

Characteristics	Utilization of SRH services		***p***-value	Crude OR (95%CI)
	Yes n (%)	No n (%)		
**Listen to radio/FM**				
Often	37(21.3)	137(78.7)	0.004*	2.3(1.303–4.088)
Never	22(10.5)	188(89.5)		Ref
**Watch TV**				
Often	32(16.0)	168(84.0)	0.719	1.1(0.635–1.932)
Never	27(14.7)	157(85.3)		Ref
**Read newspaper**				
Often	8(15.1)	45(84.9)	0.953	1.0(0.435–2.192)
Never	51(15.4)	280(84.6)		Ref
**Surf the internet to get information on health**				
Often	11(23.4)	36(76.6)	0.107	1.8(0.877–3.860)
Never	48(14.2)	289(85.8)		Ref

The crude odds ratio is the odds ratio that identifies the association between variables with the use of SRH services. The variable for which *p*-value is less than 0.05(*) is considered significant

*Ref* reference group

### Knowledge and perception of women with disabilities and utilization of SRH services

A majority (72%) of respondents had heard about SRH. Radio/FM (40%) followed by the teacher (37%) were found to be the major source of SRH related information. More than two-thirds (69%) of them had very poor knowledge of SRH (Table 9).

**Table 9:** Knowledge of respondents on SRH (*n* = 384)

Variables	Frequency	Per cent
**Heard about SRH**		
Yes	109	28.4
No	275	71.6
**Source of SRH related information (*n*** = 109)*		
Friend	24	22.0
Family Member	20	18.3
Health worker	26	23.9
Female Community Health Volunteer	26	23.9
Teacher	40	36.7
Mother’s/Women’s group	6	5.5
Training	7	6.4
Radio, FM	44	40.4
TV	23	21.1
Internet	8	7.3
Newspaper	11	10.1
Poster, Pamphlet	2	1.8
Study books	4	3.7
**Knowledge of SRH**^**a**^		
Good	4	1.0
Satisfactory	45	11.7
Poor	68	17.7
Very poor	267	69.5
**Have comprehensive knowledge about HIV**^**b**^		
Yes	2	0.5
No	382	99.5

Note: *Multiple responses

^a^Knowledge of SRH was a composite measure adapted from Measure Evaluation for Sexual and Reproductive Health Knowledge [20]

^b^Comprehensive knowledge means knowing that consistent use of condoms during sexual intercourse and having just one uninfected faithful partner can reduce the chance of getting HIV, knowing that a healthy-looking person can have HIV, and rejecting the two most common local misconceptions about transmission or prevention of HIV [31]

Those women with disabilities with knowledge of SRH (OR = 4.3; CI: 2.428–7.692), had perceived the need for SRH services (OR = 9.9; CI: 4.382–22.550), perceived themselves susceptible to SRH related disease/condition (OR = 7.5; CI: 4.001–14.220), perceived SRH related disease/ condition as severe (OR = 3.9; CI: 1.928–7.974), and reported competent to utilize SRH services (OR = 5.7; CI: 2.996–10.668) were more likely to utilize SRH services compared to their counterparts (Table 10).

**Table 10:** Knowledge and perception of respondents on SRH and their association with utilization of SRH services (*n* = 384)

Characteristics	Utilization of SRH services		***p***-value	Crude OR (95%CI)
	Yes n (%)	No n (%)		
**Knowledge on SRH**				
Good	35(29.9)	82(70.1)	0.000*	4.3(2.428–7.692)
Poor	24(9.0)	243(91.0)		Ref
**Perceived need for SRH services**				
Yes	52(27.2)	139(72.8)	0.000*	9.9(4.382–22.550)
No	7(3.6)	186(96.4)		Ref
**Perceived susceptibility to SRH related disease/condition**				
Yes	44(32.6)	91(67.4)	0.000*	7.5(4.001–14.220)
No	15(6.0)	234(94.0)		Ref
**Perceived severity of SRH related disease/condition**				
Yes	5(36.6)	26(63.4)	0.000*	3.9(1.928–7.974)
No	44(12.8)	299(87.2)		Ref
**Perceived benefit of utilization of SRH services**				
Yes	24(20.9)	91(79.1)	0.052	1.8(0.994–3.128)
No	35(13.0)	234(87.0)		Ref
**Self-efficacy/competent to utilize SRH services**				
Yes	23(41.1)	33(58.9)	0.000*	5.7(2.996–10.668)
No	36(11.0)	292(89.0)		Ref

The crude odds ratio is the odds ratio that identifies the association between variables with the use of SRH services. The variable for which *p*-value is less than 0.05(*) is considered significant

*Ref* reference group

### Contributing factors for the utilization of SRH services: access to information, perceived severity, susceptibility and benefit

Perceived severity and susceptibility are the two constructs of the Health Belief Model [22], an intrapersonal behaviour change theory designed to elucidate how beliefs predict commitment in health-protective behaviours and screenings. Perceived severity is the belief in the degree of harm from an acquired disease/harmful state as a result of a particular behaviour. While perceived susceptibility is the subjective belief that a person may acquire a disease or enter a dire state due to a particular behaviour.

The findings from the qualitative study supported the quantitative findings. Perceived severity and susceptibility contributed to the utilization of SRH services. Those women with disabilities who did not perceive themselves susceptible to SRH related disease/condition and who did not consider SRH related disease/condition as severe had less threat to SRH related disease/condition leading to lower utilization of SRH services.

*I have never used any SRH services. I have never faced any SRH related diseases. Frankly speaking, I don’t know about the consequences. With god’s mercy, I am healthy and hope to remain the same. (participant number 2, woman with disability during in-depth interview)”*

Knowledge of SRH was found to be significantly associated with the utilization of SRH services. The qualitative findings also show that those women with disabilities who had knowledge of SRH and had access to information were aware of SRH. Those who knew about the availability of SRH services and their benefits utilized the SRH services. The study also highlighted the need to increase access to SRH information by intervening at the household level.

*“Some women with disabilities have access to information on SRH through media, female community health volunteers and their neighbours/friends. They are aware of SHR services and utilize them. While other women with disabilities stay at their homes. They lack access to information, education and communication. We need to make SRH information materials available in every home of women with disabilities and also intervention at the family level. (participant number 4, local political leader during interview)”*

### Access to and utilization of SRH services

A majority (73%) of respondents reported that the nearest health facility was not disability-inclusive [11] especially referring to the inaccessible road (48%) (Table 11).

**Table 11:** Disability-friendly related characteristics of health facility (*n* = 384)

Variables	Frequency	Per cent
**The nearest health facility is disability-inclusive** [11]		
Yes	103	26.8
No	281	73.2
**The reason behind not disability-inclusive health facility (*n*** **= 281)**^**a**^		
Inaccessible road to reach the health facility	262	48.5
No ramp in the health facility	156	28.9
The room inside the health facility is not accessible	98	18.1
Bad behaviour of health workers	5	0.9
Discrimination	1	0.2
No disability-inclusive Information, Education and Communication(IEC)/ Behaviour Change Communication(BCC) materials	15	2.8
Distant health facility	3	0.6

^a^Multiple responses

Among 384 respondents, less than half (47%) could access SRH services within 30 min. The average time taken to visit the nearest health facility for SRH services was 45 min. On average, the distance between home and the nearest health facility with SRH services was nine kilometres. The quantitative study does not show any significant association between the accessibility of health facilities and the utilization of SRH services among women with disabilities (Table 12).

**Table 12:** Accessibility of health facilities and their association with the utilization of SRH services (*n* = 384)

Characteristics	Utilization of SRH services		***p***-value	Crude OR (95%CI)
	Yes n (%)	No n (%)		
**Time required to reach the nearest health facility**				
> 30 mins	32(18.0)	147(82.0)	0.204	1.4(0.822–2.504)
≤ 30 mins	27(13.2)	178(86.8)		Ref
**Distance between home and the nearest health facility**				
> 1 km	20(14.9)	114(85.1)	0.861	0.9(0.529–1.704)
≤ 1 km	39(15.6)	211(84.4)		Ref
**Enrolment to health insurance**				
Yes	13(17.1)	63(82.9)	0.639	1.2(0.599–2.307)
No	46(14.9)	262(85.1)		Ref
**The nearest health facility is disability-friendly**				
Yes	17(16.5)	86(83.5)	0.708	1.1(0.608–2.081)
No	42(14.9)	239(85.1)		Ref

The crude odds ratio is the odds ratio that identifies the association between variables with the use of SRH services. The variable for which *p*-value is less than 0.05 is considered significant

*Ref* reference group

### Structural barriers to the utilization of SRH service among women with disabilities

Access refers to ensuring persons with disabilities can access health services and facilities on an equal basis with others in a way that promotes their dignity and independence. Structural barriers are obstacles in natural or man-made environments that prevent access or hinder persons with disabilities from moving around independently [11].

Though the quantitative findings did not show the significant association between the accessibility of health facilities and utilization of SRH services among women with disabilities, the qualitative findings showed that structural barriers such as distant health facilities and lack of accessible infrastructure (road and health facility) hindered the utilization of SRH services among women with disabilities.

*“Persons with severe and profound disabilities are deprived of utilizing SRH services in remote areas as health facilities are not accessible for them and the health providers are unable to reach their home. (participant number 3, local political leader during interview)”*

### Attitudinal barriers to the utilization of SRH service among women with disabilities

One of the most significant barriers to effective participation and inclusion of persons with disabilities are negative attitudes and stereotypes. People often see persons with disabilities as incapable, dependent or weak. This perpetuates their segregation and exclusion from society [11].

The qualitative finding showed that the behaviour towards women with disabilities had changed over time. But still, stigmatization and bad behaviour of health service providers persisted.

*“The situation has been changed. Now, people treat us (women with disabilities) well. But (pause) some health workers still use inappropriate words and are rude. I feel uncomfortable expressing my concerns and query while visiting the health facility. (participant number 2, woman with disability during in-depth interview)”*

Health service providers’ attitudes that women with disabilities should not be sexually active denies them access to sexual rights and services. In this study, local leaders, health workers, female community health volunteers and women with disabilities themselves perceived that many women with disabilities might be sexually active and need SRH services. However, the study showed the practice of providing information on SRH based on the marital status of women with disabilities that highlighted the need to sensitize health service providers on the SRH needs of women with disabilities.

*“During the home visits, I provide information about family planning to married women. How can I talk about family planning and abortion service to unmarried women? (chuckle). Unmarried women with disabilities are not interested and they do not need it. (participant number 8, female community health volunteer during focus group)”*

*“Women with disabilities may have the desire to get married. But if they get married and have children, then there will be a big problem. They are unable to provide adequate care to their children. As they have an impairment, how can they raise their children properly? (participant number 7, female community health volunteer during focus group)”*

### Factors independently associated with utilization of SRH services

Fifteen characteristics that exhibited significant association with utilization of SRH services at 95% CI in bivariate analysis were further subjected to multivariate analysis. There was no problem of collinearity among independent variables as the highest variance inflation factor was 2.009.

Those women with disabilities who were ever married (AOR = 121.7, CI: 12.206–1214.338), perceived the need for SRH services (AOR = 5.5; CI: 1.419–21.357) and perceived themselves susceptible to SRH related disease/ condition (AOR = 6.0, CI:1.978–18.370) were more likely to utilize SRH services compared to their counterparts (Table 13).

**Table 13:** Factors independently associated with utilization of SRH services (*n* = 384)

Characteristics	Crude		Adjusted	
	***p***-value	OR (95%CI)	***p***-value	OR (95%CI)
**Age**				
35 and above	0.000*	3.4(1.795–6.430)	0.879	0.9(0.224–3.594)
Below 35		Ref		Ref
**Type of family**				
Joint	0.024*	2.0(1.100–3.760)	0.821	0.847(0.201–3.574)
Nuclear		Ref		Ref
**Educational status of caretaker (*n*** **= 314)**				
Literate	0.048*^#^	2.9(1.009–8.588)	0.468	1.7(0.400–7.359)
Cannot read/write		Ref		Ref
**Marital Status**				
Ever married	0.000*^#^	174.7(23.818–1281.599)	0.000*	121.7(12.206–1214.338)
Never married		Ref		Ref
**Household size**				
Less than five	0.004*	2.3(1.304–4.011)	0.459	1.7(0.434–6.352)
Five or more		Ref		Ref
**Occupation**				
Employed	0.000*	4.2(2.353–7.441)	0.130	2.5(0.762–8.236)
Unemployed		Ref		Ref
**Type of disability**				
Physical disability	0.000*	3.0(1.692–5.254)	0.818	0.9(0.232–3.169)
Other than physical		Ref		Ref
**The severity of disability (*n*** **= 318)**				
Mild	0.001*	2.8(1.500–5.262)	0.722	1.3(0.318–5.223)
Moderate/Severe/Prof.		Ref		Ref
**Women empowerment**				
Moderate and high	0.000*	4.5(2.471–8.101)	0.968	1.0(0.259–3.659)
Low empowered		Ref		Ref
**Listen to radio/FM**				
Often	0.004*	2.3(1.303–4.088)	0.738	1.2(0.351–4.389)
Never		Ref		Ref
**Knowledge on SRH**				
Good	0.000*	4.3(2.428–7.692)	0.936	0.9(0.254–3.528)
Poor		Ref		Ref
**Perceived need for SRH services**				
Yes	0.000*	9.9(4.382–22.550)	0.014*	5.5(1.419–21.357)
No		Ref		Ref
**Perceived susceptible to SRH related disease/condition**				
Yes	0.000*	7.5(4.001–14.220)	0.002*	6.0(1.978–18.370)
No		Ref		Ref
**Perceived severity to SRH related disease/condition**				
Yes	0.000*	3.9(1.928–7.974)	0.251	2.6(0.504–13.760)
No		Ref		Ref
**Self-efficacy/competent to utilize SRH services**				
Yes	0.000*	5.7(2.996–10.668)	0.891	1.1(0.255–4.813)
No		Ref		Ref

The adjusted odds ratio is the odds ratio that identifies the association between variables with the use of SRH services taking all variables into account

The variable for which *p*-value is less than 0.05(*) is considered significant

^#^Fisher’s exact test

*Ref* reference group

### Barriers to the utilization of SRH services among women with disabilities adapted from health belief model

Health Belief Model is a social psychological health behaviour change model developed to explain and predict health-related behaviours, particularly regarding the uptake of health services [22]. As Health Belief Model is the most widely used theory in health behaviour research, it was adopted to present the findings from qualitative data of the study.

There were various factors responsible for the utilization of SRH services. The first factor was an individual perception, which was related to perceived susceptibility and perceived severity. Perceived susceptibility referred to subjective assessment of the risk of developing a health problem. The combination of perceived severity and perceived susceptibility was referred to as perceived threat [22]. The study showed that those women with disabilities who did not perceive themselves susceptible to SRH related disease/condition and who did not consider SRH related disease/condition as serious were less likely to utilize SRH services. The lower perceived threat led to lower utilization of SRH services.

The modifying variables affect health-related behaviours indirectly by affecting perceived seriousness, susceptibility, benefits, and barriers [22]. Perceived severity and perceived susceptibility to SRH related disease/condition depended on knowledge of SRH. The study showed that those women with disabilities who could not read/write, belonged to low socioeconomic status, lacked information, and lack sharing among neighbours/friends were less likely to utilize SRH services.

A cue, or trigger, is necessary for promoting engagement in health-promoting behaviours [22]. The study showed that low media exposure (radio/FM, TV, internet other social media) limited engagement in health-promoting behaviours and finally resulted in low utilization of SRH services among women with disabilities.

**Fig. 1:**
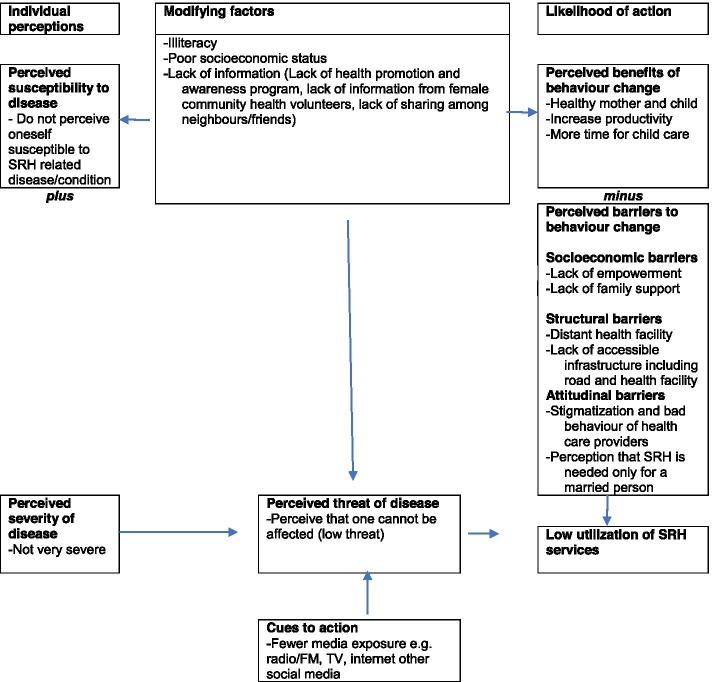
Barriers to the utilization of sexual and reproductive health services among women with disabilities adapted from Health Belief Model

According to the Health Belief Model, the likelihood of action is the result of perceived benefits minus perceived barriers [22]. The study shows that those women with disabilities who perceived the benefits of utilization of SRH services were more likely to utilize them. The perceived barriers for utilization of SRH services among women with disabilities were socioeconomic barriers (lack of empowerment, lack of family support), structural barriers (distant health facility, lack of accessible infrastructure including road and health facility) and attitudinal barriers (stigmatization, bad behaviour of health care providers, and perception that SRH is needed only for married person) (Fig. 1).

## Discussion

Our findings provide good evidence that utilization of SRH services among women with disabilities was very low in Ilam district, Nepal. Among 384 women with disabilities of reproductive age, only 15% had ever utilized any SRH services. The finding is similar to the finding from Cameroon [32], which showed that only 20% of women with disabilities had ever used SRH services.

The study showed that the educational status of women with disabilities (OR = 2.3, CI:1.201–4.612) and their caretaker (OR = 2.944, CI:1.009–8.588) were positively associated with utilization of SRH services. Education and health awareness programs were influencing factors while lack of family support and stigmatization were barriers to utilization of SRH services. The finding contrasts with the study from Cameroon, which showed that education level was not associated with the experience of difficulties in using SRH services [32]. Another study conducted in three countries (Uganda, Zambia and Ghana) showed that lack of family support and stigma related to HIV and disability hindered the utilization of SRH services [33].

The study showed a positive relationship between empowerment and the utilization of SRH services. Employed (OR = 4.184; CI:2.353–7.441) and empowered (OR = 4.5, CI:2.471–8.101) women with disabilities were more likely to utilize SRH services. However, the cross-sectional study from Cameroon [32] did not show any association between lifetime work participation and utilization of SRH services by persons with disabilities (*p* = 0.3).

The study showed that women having physical disabilities (OR = 2.982; CI:1.692–5.254) and mild disability (OR = 2.810; CI: 1.500–5.262) were more likely to utilize SRH services than their counterparts. The qualitative finding showed that women with physical disabilities could move independently using assistive devices such as crutches/wheelchairs. Those women having intellectual disabilities or severe disabilities needed someone to escort them to health facilities, which was not possible as family members themselves were busy with their household chores and livelihood related activities. The finding is similar to the study conducted in three countries (Uganda, Zambia and Ghana), which also highlighted that people with disabilities often need to travel with an assistant to help them manoeuvre around obstacles they encounter on the way. This brought additional complications due to the difficulty of finding someone prepared to give up their time and be publicly seen [33].

The study showed that women with disabilities who have knowledge of SRH (OR = 4.322; CI: 2.428–7.692) were more likely to utilize SRH services. Illiteracy and lack of information hindered the utilization of SRH services. The finding is similar to a qualitative study from Uganda [34] and a literature review conducted in developing countries [35], where lack of information was identified as one of the key barriers to access SRH services by people with disabilities.

Though Nepal has a national guideline for disability-inclusive health service [11] which guides how to mainstream disability inclusion in health service delivery, and how health providers can operationalize their responsibilities under disability-related laws and policies, its weak enforcement persists which echoes with broader studies [12, 13] related to implementation problems in Nepal’s health system. The qualitative finding showed that women with disabilities faced socioeconomic barriers (lack of empowerment, lack of family support), structural barriers (distant health facility, inaccessible infrastructure including road and health facility), and attitudinal barriers (stigmatization and bad behaviour of health care providers, perception that SRH is needed only for married) to access of SRH services. Noticeably, these barriers are echoed by several studies from Nepal among the population (without disability) [36–38] and therefore women with disabilities inevitably add a layer of barriers. The findings are consistent with the findings from Uganda [34] where negative attitudes of service providers, distant health facilities and unfriendly physical structures were identified as barriers to access SRH services by persons with disabilities. Moreover, long queues at health facilities and high costs of services involved were also identified as barriers to access SHR services. However, this study does not support the findings.

Studies from Uganda [34] and India [39] showed that people perceived persons with disabilities as asexual, which was identified as one of the barriers to access SRH services. This study contrasts with those findings. In this study, local leaders, health workers, female community health volunteers and women with disabilities themselves perceived that many women with disabilities might be sexually active and need SRH services. However, the qualitative study showed the practice of providing information on SRH based on the marital status of women with disabilities.

The findings of the study are also similar to a literature review of barriers to healthcare services for people with disabilities in developing countries [35], where inaccessible facilities, limited mobility, stigmatization and staff attitude were identified as barriers to access healthcare services by persons with disabilities. Moreover, other barriers such as additional costs of healthcare and communication barriers were also identified. However, this study does not support the findings.

This study highlighted the importance of individual perception for the utilization of SRH services among women with disabilities. Individual perception such as the perceived need for SRH services (AOR = 5.505; CI: 1.419–21.357) and perceived susceptibility to SRH related disease/ condition (AOR = 6.028, CI: 1.978–18.370) were found to be positively associated with utilization of SRH services among women with disabilities.

Though SRH services should be provided irrespective of one’s marital status, the study showed that those ever-married women with disabilities were more likely to utilize SRH services compared to their counterparts (AOR = 121.7, CI: 12.206–1214.338). The qualitative findings also showed that health service providers hesitated to talk about SRH services to unmarried women with disabilities.

These findings call for a need to promote awareness-raising programs to women with disabilities and their family members, sensitize health service providers on SRH needs of women with disabilities and ensure the provision of disability-inclusive SRH services in all health facilities.

### Limitations of the study

The study was conducted in one of the hilly districts of province number one of Nepal, so it does not represent the national scenario. Furthermore, because of its cross-sectional design, causality cannot be established. Although the sample size in this study was based on the standard calculation, a few sub-samples representing variables in the regression models had low numbers and may have generated a high standard error. Future studies with a large sample size for all sub-variables can make a robust statistical assessment. Moreover, current trends of SRH was not under the scope of the study. Likewise, the themes were pre-determined from the Health Belief Model for the qualitative arm of the study. However, after categorizing the data using the deductive approach, additional categories and themes were added, inductively, to incorporate the emerging themes from the transcripts.

## Conclusion

The utilization of SRH services among women with disabilities was very low (15%) in Ilam district, Nepal. Among 121 women with disabilities who had ever experienced pregnancy and childbirth, only half (51%) had received antenatal checkups, more than two-thirds (67%) had delivered their child at home mainly with support from family members (52%) and only 34% had received postnatal care. No requirement (57%) and being unaware of SRH services (24%) were the main reasons for not utilizing SRH services. A majority (81%) of them reported that the nearest health facility was not disability-inclusive (73%) specifically referring to the inaccessible road (48%). SRH services were mostly utilized by those women with disabilities who were married, who felt the need for SRH services and felt susceptible to SRH related disease/conditions. Qualitative findings revealed that illiteracy, poor socioeconomic status, and lack of information inhibited the utilization of SRH services. Furthermore, the study showed that women with disabilities faced socioeconomic barriers (lack of empowerment, lack of family support), structural barriers (distant health facility, inaccessible infrastructure including road and health facility), and attitudinal barriers (stigmatization and bad behaviour of health care providers, perception that SRH is needed only for married) to access SRH services. These findings call for a need to promote awareness-raising programs to women with disabilities and their family members, sensitize health service providers on SRH needs of women with disabilities, and ensure the provision of disability-inclusive SRH services in all health facilities.

## Supplementary Information

**Additional file 1.**

## Data Availability

The datasets analyzed during the current study are available from the corresponding author on reasonable request.

## Abbreviations

AOR: Adjusted Odds Ratio
CDPS: Central Department of Population Studies
CI: Confidence Interval
CRPD: Convention on the Rights of Persons with Disabilities
FP: Family Planning
GBV: Gender-Based Violence
HIV: Human Immunodeficiency Virus
NDHS: Nepal Demographic and Health Survey
OR: Odds Ratio
SD: Standard Deviation
SDGs: Sustainable Development Goals
SPSS: Statistical Package for Social Science
SRH: Sexual and Reproductive Health
STIs: Sexually Transmitted Infections
UN: United Nations
WHO: World Health Organization
WwDs: Women with Disabilities
