# Supplementary material for: Factors affecting utilization of sexual and reproductive health services among women with disabilities- a mixed-method cross-sectional study from Ilam district, Nepal
Source: BMC Health Serv Res. 2021 Dec 23;21:1361. doi: 10.1186/s12913-021-07382-4 (PMC8705122; doi:10.1186/s12913-021-07382-4)
Supplement: Supplementary file 1 — Additional file 1. [file 12913_2021_7382_MOESM1_ESM.pdf]

Types and severity of disabilities

In the study, types and severity of disabilities were classified into 10 types and four categories as per the Nepal disability rights act 2017 [1].

Types of disabilities

1. Physical disability: the problem that arises in the operation of physical parts, use and movement due to problems in nerves, muscles and composition and operation activities of bones and joints (e.g., the disability that arises due to polio, lack of a physical organ, the effect of leprosy, muscular dystrophy, the permanent problem associated with joints and backbone, reversal of clubfeet, the problem associated with rickets bones), and a person whose height at sixteen years of age is excessively lower than the average height
2. Disability-related to vision: the condition where there is no knowledge about an object's figure, shape, form and colour due to the following problem with the vision: (a) Blindness: a person who cannot distinguish fingers of hand by both eyes from a ten feet distance or who cannot read the letters on the fourth row of the Snellen chart (3/60), even upon utilization of medicines, operation, lenses or lens. (b) Low vision: a person who cannot distinguish fingers of hand by both eyes from a twenty feet distance or who cannot read the letters on the fourth row of the Snellen chart (6/18), even upon utilization of medicines, operation, lenses or lens. (c) Total absence of sight: a person who cannot differentiate brightness or darkness.

3. Disability-related to hearing: problems arising in an individual who cannot discriminate composition of the parts of hearing and voice, rise and fall, and level and quality of voice, (a) Deaf: a person who cannot hear a voice above eighty decibels or who needs sign language for communication. (b) Hard of hearing: a person who needs a hearing device to hear or who can hear a voice from sixty-five to eighty decibels.
4. Deaf-Blind: a person who can neither hear nor see.
5. Disability-related to voice and speech: difficulty produced in parts related to voice and speech e.g. difficulty in rising and fall of voice to speak, unclear speech, repetition of words and letters.
6. Mental or psychosocial disability: the inability to behave by age and situation and delay in intellectual learning and performing intellectual activities e.g. problems arising in the brain and awareness, orientation, alertness, memory, language, and calculation.
7. Intellectual disability: a person has a problem in doing activity relative to the age or environment due to lack of intellectual development in regards to his/her chronological age (for example, Down syndrome.)
8. Disability associated with haemophilia: a person who has a problem in the clotting of blood due to deflection in clotting factors in the blood as a genetic effect.
9. Disability-related to Autism: a person who has a problem in the development of veins or tissues and functionality (e.g., a person who has difficulty communicating, understanding and applying general social rules, and who

does not show normal behaviour along with the age, who shows an abnormal reaction, repeats the same activity, does not assimilate with others or makes reaction instantly).

10. Multiple disabilities: a person who has a problem of two or more types of disability mentioned above (e.g., cerebral palsy).

Severity of disability

1. Profound disability: a person who has difficulty performing his or her day-to-day activities even with the continuous support of others.
2. Severe disability: a person who needs the continued support of others to perform personal activities and involvement in social activities.
3. Moderate disability: a person who can regularly participate in his or her daily activities and social activities if the physical facility is available, an environmental barrier is ended or education/training is provided.
4. Mild disability: a person who can regularly participate in his or her daily activities and social activities if there exists no physical and environmental barrier

Reference

1. Nepal Law Commission. Nepal disability rights act 2017. Kathmandu, Nepal: Government of Nepal; 2017.
